# *In Vitro* Phenotypic, Genomic and Proteomic Characterization of a Cytokine-Resistant Murine β-TC3 Cell Line

**DOI:** 10.1371/journal.pone.0032109

**Published:** 2012-02-29

**Authors:** Antonina Coppola, Laura Tomasello, Giuseppe Pizzolanti, Ida Pucci-Minafra, Nadia Albanese, Gianluca Di Cara, Patrizia Cancemi, Maria Pitrone, Alessandra Bommarito, Elvira Carissimi, Giovanni Zito, Angela Criscimanna, Aldo Galluzzo, Carla Giordano

**Affiliations:** 1 Section of Endocrinology, Dipartimento Biomedico di Medicina Interna e Specialistica (DIBIMIS), University of Palermo, Palermo, Italy; 2 Centro di Oncobiologia Sperimentale (COBS), University of Palermo, Palermo, Italy; 3 Department of Physics, Centro di Oncobiologia Sperimentale (COBS), University of Palermo, Palermo, Italy; 4 Section of Experimental Oncology, Dipartimento Biomedico di Medicina Interna e Specialistica (DIBIMIS), University of Palermo, Palermo, Italy; 5 Institute of Biomedicine and Molecular Immunology “A. Monroy” (CNR – IBIM), Palermo, Italy; Universita Magna-Graecia di Catanzaro, Italy

## Abstract

Type 1 diabetes mellitus (T1DM) is caused by the selective destruction of insulin-producing β-cells. This process is mediated by cells of the immune system through release of nitric oxide, free radicals and pro-inflammatory cytokines, which induce a complex network of intracellular signalling cascades, eventually affecting the expression of genes involved in β-cell survival.

The aim of our study was to investigate possible mechanisms of resistance to cytokine-induced β-cell death. To this purpose, we created a cytokine-resistant β-cell line (β-TC3R) by chronically treating the β-TC3 murine insulinoma cell line with IL-1β + IFN-γ. β-TC3R cells exhibited higher proliferation rate and resistance to cytokine-mediated cell death in comparison to the parental line. Interestingly, they maintained expression of β-cell specific markers, such as PDX1, NKX6.1, GLUT2 and insulin. The analysis of the secretory function showed that β-TC3R cells have impaired glucose-induced c-peptide release, which however was only moderately reduced after incubation with KCl and tolbutamide. Gene expression analysis showed that β-TC3R cells were characterized by downregulation of IL-1β and IFN-γ receptors and upregulation of SOCS3, the classical negative regulator of cytokines signaling. Comparative proteomic analysis showed specific upregulation of 35 proteins, mainly involved in cell death, stress response and folding. Among them, SUMO4, a negative feedback regulator in NF-*k*B and JAK/STAT signaling pathways, resulted hyper-expressed. Silencing of SUMO4 was able to restore sensitivity to cytokine-induced cell death in β-TC3R cells, suggesting it may play a key role in acquired cytokine resistance by blocking JAK/STAT and NF-*k*B lethal signaling.

In conclusion, our study represents the first extensive proteomic characterization of a murine cytokine-resistant β-cell line, which might represent a useful tool for studying the mechanisms involved in resistance to cytokine-mediated β-cell death. This knowledge may be of potential benefit for patients with T1DM. In particular, SUMO4 could be used as a therapeutical target.

## Introduction

Type 1 diabetes mellitus (T1DM) is an autoimmune disease characterized by a strong inflammatory response. The immune reaction against β-cells, i.e. insulitis, precedes β-cell destruction and overt disease onset. This complex process is mediated by a cellular component – that includes macrophages, neutrophils, CD4^+^ and CD8^+^ T cells - and a molecular component – represented mainly by pro-inflammatory cytokines, free oxygen and nitric oxide radicals [1]–[3]. All these elements act in concert in initiating and maintaining β-cell destruction. In particular, tumor necrosis factor-α (TNF-α), interleukin-1β (IL-1β), inteleukin-6 (IL-6), and interferon-γ (IFN-γ) activate a complex network of intracellular signalling cascades, which induce necrosis or apoptosis [4], [5]. IL-1β and TNF-α activate nuclear factor kappa B (NF-*k*B), the mitogen-activated protein kinases p38 and c-Jun N-terminal kinase; while IFN-γ acts via the Janus Kinase (JAK)/signal transducer and activator of transcription-1 (STAT-1) pathway [6]–[9]. In animal models, neutralisation of IL-1β and IFN-γ signalling appears to protect against T1DM [10]–[12].

Sensitivity to cytokine, free radicals and toxic chemicals is exclusive of the β-cell. Prolonged exposure to cytokine severely suppresses its function, eventually leading to T1DM. However, the exact molecular mechanisms and pathways involved are still uncertain [13]. When islets and β-cells are exposed to cytokines, multiple changes in the expression profiles of mRNA and proteins are detectable. These changes comprise up- and downregulation as well as *de novo* synthesis of several groups of genes, which contribute to the loss of differentiated β-cell functions and trigger both pro- and anti-apoptotic mechanisms. Decreased insulin production [14], [15] and reduced growth capacity of cytokine-exposed β-cells or islets [16] have been also described. However, interpretation of these data remains uncertain due to the difficulty in discriminating between early “primary” and late “secondary” effects of cytokine exposure.

The aim of our study was to analyze possible mechanisms involved in resistance to cytokine-induced β-cell death. To this purpose, we first exposed the mouse insulinoma cell line β-TC3 to chronic treatment with IL-1β + IFN-γ, generating a cytokine-resistant cell line (β-TC3R). β-TC3R cells maintained the expression of specific markers and the secretory machinery typical of mature β-cells, although with consistently lower glucose-induced insulin secretion (measured as c-peptide) compared to parental β-TC3 cells. Analysis of both protein and gene expression profiles showed upregulation of 35 proteins in β-TC3R, among them the Suppressor of Cytokine Signaling 3 (SOCS-3) and the Small Ubiquitin-related Modifier 4 (SUMO4). The latter, was able to restore sensitivity to cytokine-induced cell death in β-TC3R cells after silencing, suggesting it could be potentially used as a therapeutic target.

## Materials and Methods

### Cell lines and culture conditions

Mouse β-TC3 cell line derived by primary culture of insulinoma was kindly provided by S. Efrat (Albert Einstein College of Medicine, NY) [17]. Cells were cultured in high glucose DMEM, 15% heat-inactivated fetal bovine serum (FBS), 1% L-glutamine and 1% antibiotics, at 37°C and 5% CO_2_.

### Selection of cytokine-resistant β-TC3R cell line

Cytokine-resistant β-TC3R cell line was obtained exposing β-TC3 cells for 12 weeks to increasing concentrations of recombinant mouse IFN-γ and IL-1β (PeproTech). The multi-step selection process was started by incubating β-TC3 cells for 48 hours in culture medium supplemented with 10 IU/ml IL-1β and IFN-γ. Cells were then cultured without cytokines for additional 48–72 hours to allow growth of surviving resistant cells. Concentration of cytokines (single IL-1β and IFN-γ or combination of both) was then gradually increased (50, 100 and 250 IU/ml) to further select β-TC3R cells.

### Cell proliferation, viability, cell cycle and apoptosis of β-TC3 and β-TC3R cells

Cell proliferation was assessed by colorimetric assay using 3-(4,5-Dimethylthiazol-2-yl)-2,5-diphenyltetrazolium bromide (MTT). Both β-TC3 and β-TC3R cells were plated in a 96-well plate with 100 µL medium/well and cultured up to 96 hours. Proliferation rate was evaluated by UV absorption spectrum at 550 nm, after MTT incubation for 4 hours at 37°C.

Viability was also assessed by MTT, culturing cells with media containing 100 IU/ml IL-1β or 100 IU/ml IFN-γ, or both cytokines for 24, 48 and 72 hours. The reduction in optical density (OD) caused by cytotoxic effect of cytokines was used as a measurement of cell viability.

Cell cycle of β-TC3 and β-TC3R cells was analyzed by flow cytometry (FACSCalibur, Becton Dickinson), after treatment with cytokines (100 IU/ml) for 72 hours. Cells suspension was fixed in 70% ethanol and stained with propidium iodide for analysis.

Apoptosis was evaluated by Caspase 3 assay and DNA-laddering. For Caspase 3 assay, cells were first fixed and permeabilized with Cytofix-Cytoperm kit (BD Pharmingen), incubated with monoclonal IgG rabbit anti-Active Caspase 3 (BD Pharmingen) and fluorescein isothiocyanate (FITC)-conjugated polyclonal goat anti-rabbit IgG (Santa Cruz Biotechnology), according to the manufacturer's instructions. Data were analyzed with CELLQuest Pro software (Becton Dickinson). Gating was implemented based on negative control staining profiles.

For DNA-laddering, β-TC3 and β-TC3R cells were cultured in medium containing cytokines (100 IU/ml) for 72 hours at a cell density of 1×10^6^, then scraped and lysed at 37°C for 2 hours in buffer containing 10 mM Tris-HCl (pH 8.0), 0.1 M EDTA (pH 8.0), 0.5% SDS, and 20 µg/ml RNase (DNase-free). Cell lysates were treated with proteinase K at 100 µg/ml at 57°C overnight. Cellular genomic DNA was precipitated with 5 M NaCl and isopropanol for 30 minutes at −20°C. The precipitate was washed in 70% ethanol and resuspended in 10 µl Tris/EDTA. DNA (10 µg) was resolved by electrophoresis in 2% agarose gel and stained in 5 µg/ml ethidium bromide.

### Immunofluorescence

Cells were cultured in culture-slides (BD Biosciences), fixed for 15 minutes at room temperature in 2% paraformaldehyde, permeabilized with 0.1% Triton X-100/PBS (Sigma-Aldrich), washed in PBS and blocked for 30 minutes in 3% BSA/PBS. Primary antibodies were incubated for 24 hours at 4°C, while secondary antibodies were incubated for 1 hour at room temperature. The sources of antibodies and dilutions used are summarized in Table S1. Images were acquired with DM IRB inverted microscope equipped with DC300F digital camera system. All fields are representative of at least five different experiments.

### C-peptide release assays (static incubation)

After discarding culturing media, both β-TC3 and β-TC3R cells were washed several times and then incubated for 1 hour in Krebs-Ringer solution with bicarbonate and HEPES (KRBH), followed by 1 hour incubation in KRBH containing 2 mM D-glucose (basal condition). Cells were then incubated for another 1 hour in stimulating conditions with either 20 mM D-glucose, 100 µM Tolbutamide or 30 mM KCl (all from Sigma-Aldrich). Plates were incubated at 37°C on a rotating shaker and the supernatant was sampled at basal conditions and at the time points 2′, 5′, 10′, 15′, 30′, 45′ and 60′ after stimulation. C-peptide release was assessed with Rat/Mouse C-peptide 2 kit (Millipore). Data are representative of three independent experiments.

### Protein extraction

β-TC3 and β-TC3R cells were scraped and incubated in ice for 30 minutes with RIPA buffer (50 mM Tris-HCl, pH 7.4, 150 mM NaCl, 1% Nonidet P40) and protease inhibitor cocktail (Roche). Total cellular lysate was centrifuged at 14,000 rpm for 1 hour to clear cell debris, and the supernatant was dialyzed against ultrapure distilled water, lyophilized, and stored at −80°C until analysis. Protein concentration in the cellular extracts was determined using Bradford assay.

### Proteomic analysis

Proteomic preparation was performed as previously described [18]. Briefly, 40 µg of β-TC3 and β-TC3R protein lysates were separated by pH 4–10 and molecular weight 8–150 kDa. Silver stained gels were analyzed with ImageMaster 2D Platinum software. Quantitative variations in protein expression levels were calculated as the volume of the spots (i.e. integration of optical density over the spot area). To correct for differences in gel staining, spot volumes relative to the sum of the volume of all spots on each gel (%Vol), were calculated by the software. Protein identification was performed by Mass spectrometry on a Voyager DE-PRO mass spectrometer (Applied-Biosystems) after in-gel digestion of protein spots, using sequencing-grade trypsin (20 **µ**g/vial). Matrix was 2,5-Dihydroxybenzoic acid from Fluka (Sigma-Aldrich). Mass spectra were recorded in the 500–5000 Da range, using a minimum of 100 shots of laser per spectrum. Delayed extraction source and reflector equipment allowed sufficient resolution to consider MH^+^ of monoisotopic peptide masses. Internal calibration was done using trypsin autolysis fragments at m/z 842.5100, 1045.5642 and 2211.1046 Da. Peptide mass fingerprinting was compared to the theoretical masses from the SwissProt or NCBI sequence databases using Mascot (http://www.matrixscience.com).

### Western blot

Proteins were denatured in Laemmli sample buffer (2% SDS, 10% glycerol, 5% 2-mercaptoethanol, 62.5 mM Tris-HCl pH 6.8, 0.004% bromophenol blue), separated on 12% polyacrylamide gels, transferred to nitrocellulose membranes (TransBlot Transfer Medium Biorad), and blotted with the primary antibodies listed in Table S1. Antigen-antibody complexes were visualized using SuperSignal West Femto Maximum Sensitivity Substrate (Pierce) on a CCD camera (Chemidoc, Biorad). For signal detection, ECL Plus (Amersham Bioscience) reagent was used. Western blot bands were quantified by densitometry using a scanner and the associate Quantity One software (Bio-Rad). Only the above-noise values (i.e. the bands) were quantified and expressed as “adjusted volume OD”.

### Isolation of total RNA, RT-PCR and qRT-PCR

Total RNA was extracted and purified from cultured β-TC3 and β-TC3R cell lines using RNeasy Mini Kit (Qiagen, Milan, Italy) including a digestion step with DNase I set. RNA quantity and quality were assessed by UV spectrophotometry. 2 µg total RNA was reverse transcribed in a volume of 20 µl with Oligo dT primers (Applied Biosystems) and Stratascript RT (Stratagene), according to the manufacturers' protocol. Fas (TNF Superfamily, member 6), Trail (Tumor Necrosis Factor Related Apoptosis Inducing ligand), iNOS (Induced Nitric Oxide Synthase), MnSOD (Manganese Superoxide Dismutase) were analyzed by polymerase chain reaction (PCR). SOCS-3, IL-1RI, IFN-γR and SUMO4 expression was analyzed by real-time quantitative PCR (qRT-PCR) in individual samples. Total 2 ng were used to measure mRNA levels relative to GAPDH mRNA expression. Primer pair sequences, cDNA fragment sizes and annealing temperatures are listed in Table S2. All reactions were performed using a LightCycler (Roche Diagnostics GmbH, Germany). All data were analyzed with qBASE Browser, which employs a Δ-Ct relative quantification model with PCR efficiency correction and single reference gene normalization (GAPDH).

### siRNA transfection

SUMO4 was silenced using SUMO4 siRNA Duplex Oligoribonucleotides and scrambled siRNA (Non-Targeting siRNA, Invitrogen). β-TC3R cells were transfected using INTERFERin transfection agent (Polyplus Transfection), according to the manufacturer's instructions. Briefly, cells were seeded into six-well plates at a density of 2.5×10^5^ cells/well. The transfection agent and siRNA (100 nM) complex were added to the cells and incubated for 72 hours. Each assay was performed in duplicate in at least five independent experiments. Cell viability after silencing was evaluated by MTT-assay.

## Results

### Selection of a β-TC3 cell population resistant to cytokine-mediated cytotoxicity

**Figure 1:**
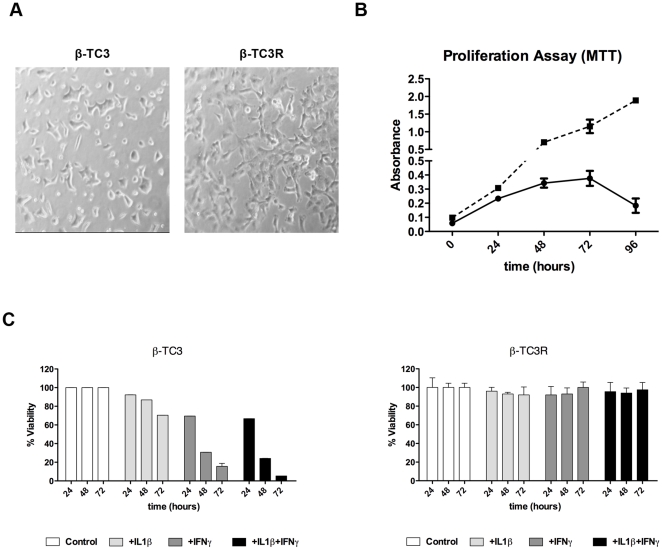
Selection of a β-TC3 cell population resistant to cytokine-mediated cytotoxicity. (**A**) Cell phenotypes: β-TC3R cells (right) show a more elongated (fibroblastic-like) appearance in comparison to parental β-TC3 cells (left). (**B**) Proliferation assay (MTT) at 0, 24, 48, 72 and 96 hours for β-TC3 (continuous line) and resistant β-TC3R (dotted line). (**C**) Cytotoxicity assay (MTT) in β-TC3 (left) and β-TC3R (right) after cytokine treatment (100 IU/ml IL-1β, 100 IU/ml IFN-γ or their combination, for 72 hours).

Cytokine-resistant β-TC3 population (β-TC3R) was obtained from a parental line that was cultured in medium with increasing concentrations of IFN-γ and IL-1β cytokines (from 10 IU/ml to 250 IU/ml) over a 12-week period. During expansion, resistance was maintained by adding repeatedly cytokines to fresh medium every 24 hrs (100 IU/ml single cytokine or combination of both). Resistant clones grew well also with higher cytokines concentrations (up to 250 IU/ml). When compared to parental β-TC3, β-TC3R showed a more fibroblastic-like morphology (Fig. 1A) and significantly increased proliferation at 24, 48, 72 and 96 hours (Fig. 1B) (24 hrs: β-TC3R 0.3±0.02 *vs* β-TC3 0.2±0.018, p = ns; 48 hrs: β-TC3R 0.68±0.02 *vs* β-TC3 0.28±0.01, p<0.001; 72 hrs: β-TC3R 1.15±0.05 *vs* β-TC3 0.35±0.06, p<0.001; 96 hrs: β-TC3R 1.85±0.03 *vs* β-TC3 0.15±0.04, p<0.001). Viability was investigated using MTT assay. As expected, sensitive β-TC3 cells showed time-dependent cytokine killing profile. After 72 hours of exposure with either IL-1β or IFN-γ viability decreased up to 75.6±1.3% and 15±2.2% respectively, while IL-1β + IFN-γ combination caused a further reduction to 8±3.2%, showing a synergistic cytotoxic effect. By contrast, β-TC3R cells maintained 100% of viability after 72 hours in the same conditions (single or combined cytokines) (Fig. 1C). Taken together, these results suggest that β-TC3 and β-TC3R cells are characterized by different proliferation rates in response to cytokine treatment.

### Evaluation of cytokine-mediated apoptosis in β-TC3 and β-TC3R cells

**Figure 2:**
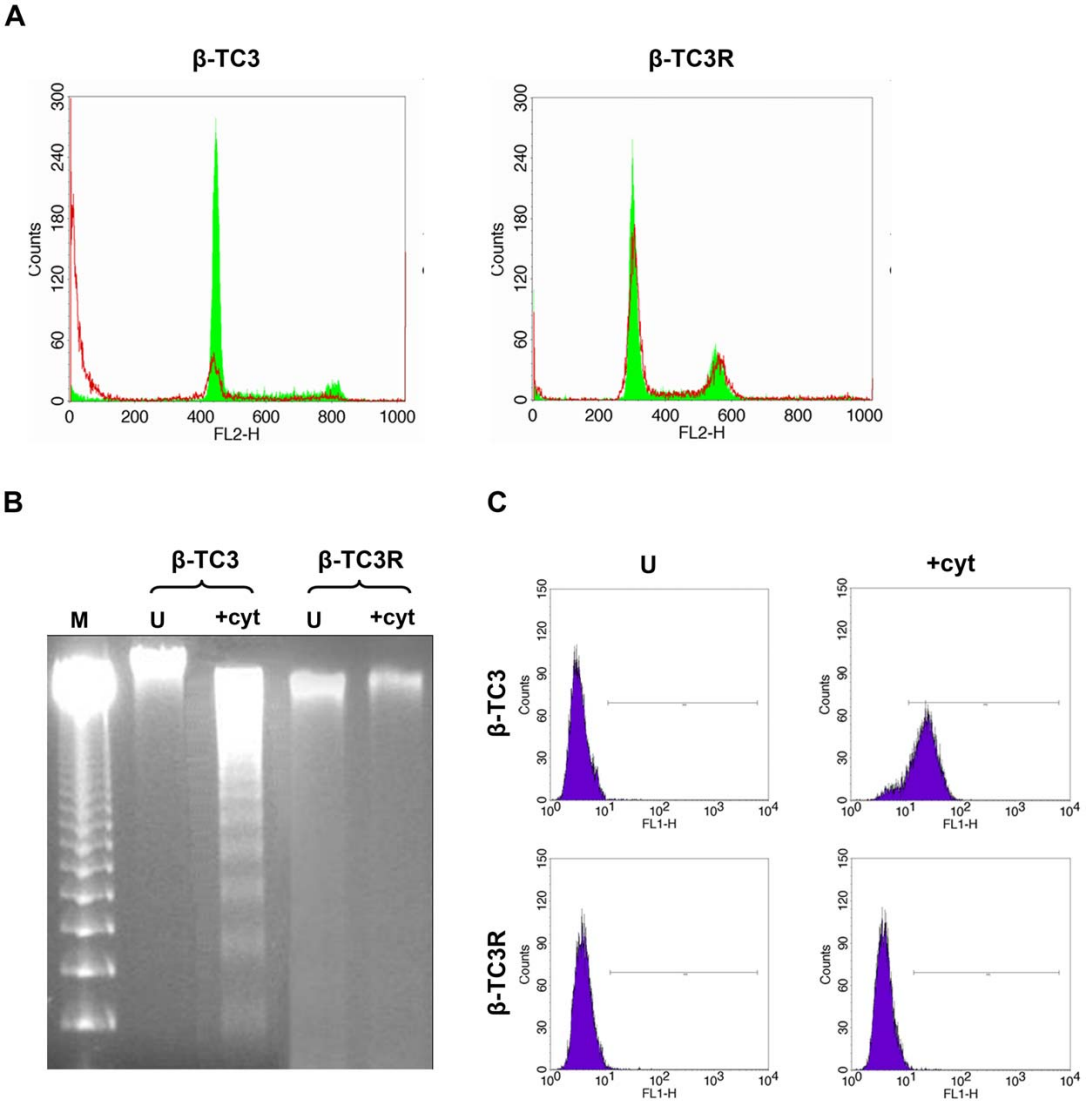
Evaluation of cytokine-mediated apoptosis in β-TC3 and β-TC3R cells. (**A**) Flow cytometry analysis of cell-cycle in β-TC3 and β-TC3R cells. Green line indicates cycle profile in untreated cells, while red line indicates cell cycle after treatment with cytokines. (**B**) Effects of cytokines on DNA fragmentation in β-TC3 and β-TC3R cells. M = DNA laddering Marker (100 bp). (**C**) Flow cytometry analysis of Caspase 3 in β-TC3 and β-TC3R cells after treatment with cytokines. No significant change was observed in β-TC3R cells. Cytokine treatment is 100 IU/ml of IL-1β + IFN-γ for 72 hours. U = untreated.

Propidium iodide analysis by flow cytometry was carried on untreated and cytokine-treated cells. After 72 hours, β-TC3 cells showed hypodiploidy, indicating DNA degradation and cell death (untreated: 10.25±0.31%; cytokine-treated: 71.8±0.63%), while in β-TC3R cells the treatment produced no significant increase in subG0 peak (untreated: 1.5±0.002%; cytokine-treated cells: 2.2±0.003%). A representative experiment is showed in Fig. 2A.

DNA fragmentation (degradation into multiples of 180-base pair long fragments), a typical hallmark of apoptosis, was analyzed by DNA laddering on agarose gels and presence of sub-diploid cells was demonstrated by flow cytometry. β-TC3 cells, cultured with combination of the two cytokines as described before, showed the typical DNA ladder on agarose gels. No DNA fragmentation was visible when β-TC3R cells were treated with the same conditions (Fig. 2B).

### IL-1β + IFN-γ cocktail does not induce cleaved Caspase 3 in β-TC3R cells

Caspase 3 activity represents a marker of the apoptotic cascade activation. Baseline Caspase 3 activity was 0.22±0.06% and 0.23±0.03% in β-TC3 and β-TC3R cells, respectively (p = NS). In β-TC3, the exposure to IL-1β + IFN-**γ** for 72 hours increased Caspase 3 levels up to 88.3±0.3%. By contrast, the same treatment in β-TC3R cells had no effect (Fig. 2C).

### Maintenance of β-cell specific markers in β-TC3R cells

**Figure 3:**
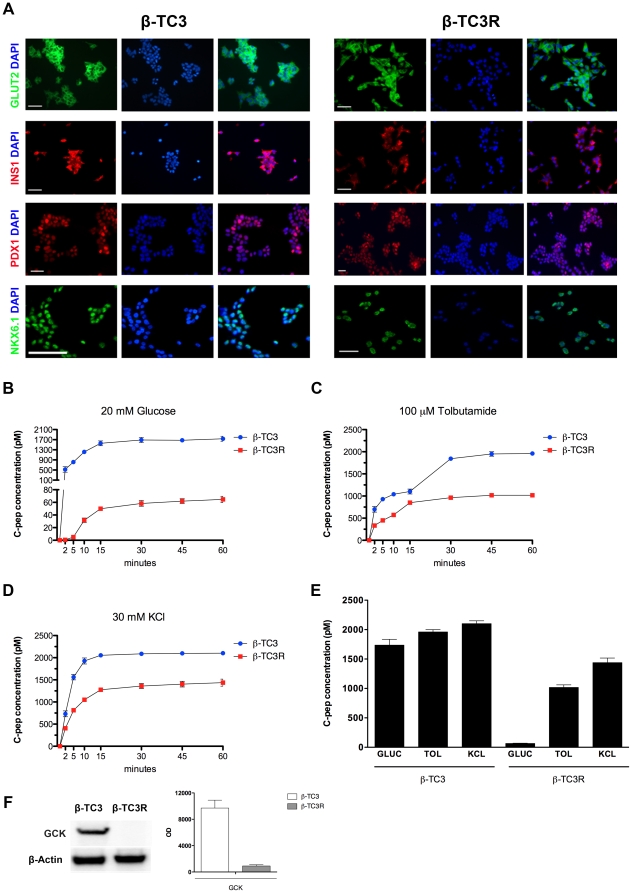
Evaluation of β-cell specific markers and secretory function in β-TC3R cells. (**A**) Immunofluorescence analysis shows persistence of β-cell markers in β-TC3R cells (right panel) despite the acquisition of cytokine resistance. Left panel: sensitive β-TC3. (**B–D**) Stimulation with 20 mM glucose (GLUC), 100 µM of the secretagogue tolbutamide (TOL) or direct depolarization with 30 mM potassium chloride (KCl) during 2-h static incubation of both β-TC3 and β-TC3R cells. (**E**) C-peptide secretion after 1-hr static incubation in β-TC3 and β-TC3R cells. (**F**) western blot analysis of glucokinase (GCK) in β-TC3 and β-TC3R cells. Graph shows quantification of proteic bands performed by densitometric analysis.

To investigate whether induced cytokine-resistance in β-TC3R cells caused a loss of β-cell specific markers, we performed immunofluorescence analysis for PDX-1 (Pancreatic Duodenal Homeobox-1), NKX6.1 (member of NK2 family of homeoprotein transcription factors), GLUT2 (Glucose transporter 2), and insulin, in both β-TC3 and β-TC3R cells (Fig. 3A). Immunostaining revealed comparable expression of the markers in both sensitive and resistant cells. The maintenance of insulin and GLUT2 (a member of the β-cell specific glucose sensor) and the nuclear localization of the two transcriptions factors PDX-1 and NKX6.1, strongly suggested the persistence of β-cell phenotype, despite the acquired cytokine resistance.

### Evaluation of the secretory capacity in β-TC3R cells

To compare the insulin secretion properties of the “native” and cytokine resistant cells, we investigated the ability to secrete C-peptide in response to several secretory stimuli. Stimulation with 20 mM glucose, 100 µM of the secretagogue tolbutamide or direct depolarization with 30 mM potassium chloride (KCl) showed that β-TC3R cells respond to the different stimuli in a regulated manner, although at a lower level in comparison to β-TC3 (Figure 3B–E). In particular, the selective impairment of glucose-induced insulin secretion could be attributed to defects in glucokinase expression, as shown by western blot analysis (Figure 3F). By contrast, incubation with tolbutamide and KCl showed only moderately reduced c-peptide levels.

### Molecular characterization of β-TC3R cells

**Figure 4:**
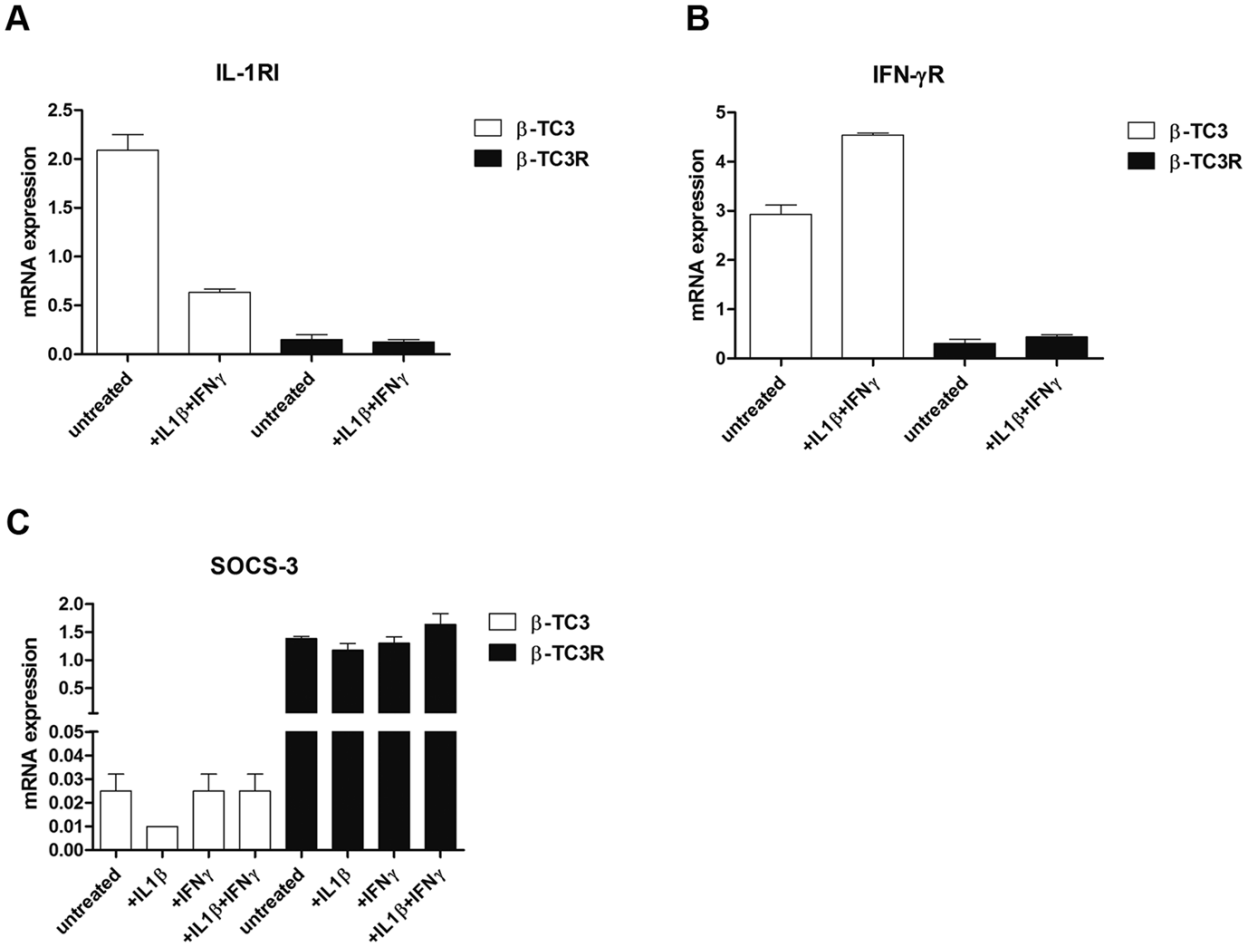
Molecular characterization of β-TC3R cells. qRT-PCR analysis of (**A**) IL-1RI, (**B**) IFN-γR, and (**C**) SOCS-3 in β-TC3 and β-TC3R cells after cytokine treatment compared to respective untreated cells. IL-1RI and IFN-γR are markedly downregulated, while SOCS-3 shows a 65-fold increase in β-TC3R compared to β-TC3 cells. Cytokine treatment is 100 IU/ml IL-1β, 100 IU/ml IFN-γ or their combination, for 72 hours.

We analyzed the expression of genes encoding interleukin-1 receptor type 1 (IL-1RI) and interferon-γ receptor (IFN-γR) in β-TC3 and β-TC3R cells. In β-TC3R cells, we found a marked decrease in mRNA levels for both receptors (96±0.8% and 89.5±1.5% decrease for IL-1RI and IFN-γR, respectively) (Fig. 4A and B). However, this observation alone cannot entirely justify the acquired resistant phenotype, as many cellular types are fully responsive to cytokines although expressing only a few copies of their receptors [19]. Therefore, we assumed other players must have been involved in the mechanism of resistance. We thus investigated members of the SOCS family, specifically SOCS-1, -2 and -3, which are known to be negative regulators of cytokine signaling. In turn, the expression of these genes is induced by various cytokines, including IL-1β and IFN-γ and is involved in inhibiting the JAK/STAT and NF-*k*B signaling pathways [20]. Although we were not able to detect any difference in SOCS-1 and -2 mRNA between β-TC3 and β-TC3R (data not shown), we found that SOCS-3 was hyperexpressed specifically in β-TC3R (Fig. 4C). In particular, after exposure to cytokines for 72 hours, β-TC3 cells showed a 1.6-fold increase of SOCS-3 mRNA level compared to untreated cells. By contrast, β-TC3R cells exhibited a 65-fold increase vs β-TC3 cells in both basal conditions and after cytokine treatment (0.015±0.009 in β-TC3 and 1.395±0.08 in β-TC3R, respectively; p<0.005). Western Blot analysis for SOCS-3 confirmed these results (data not shown).

### Proteomic profile of β-TC3 and β-TC3R cells

**Figure 5:**
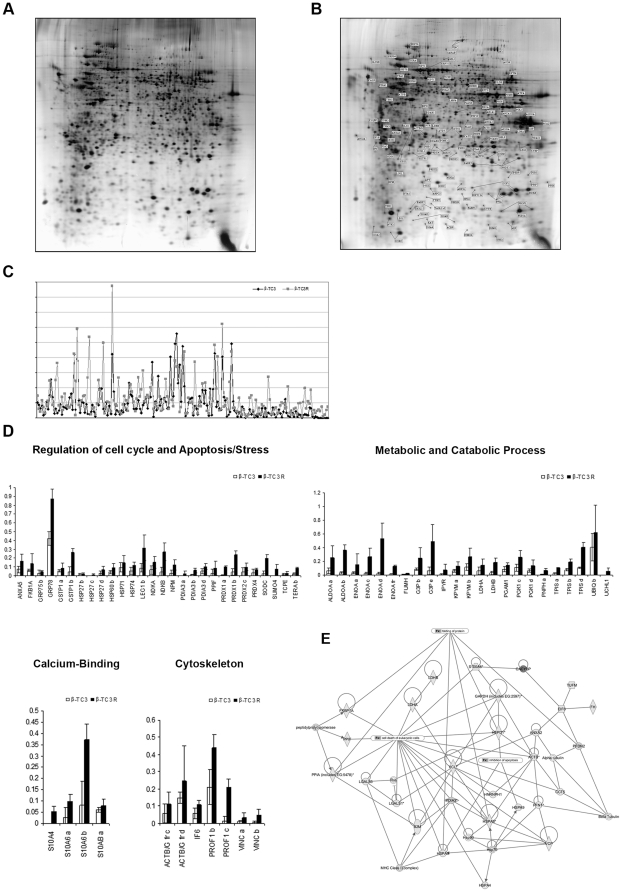
Proteomic analysis of β-TC3 and β-TC3R cells. (**A–B**) Proteomic maps. β-TC3R gel (**B**) displays the locations of the proteins that are differentially expressed in comparison to β-TC3; *see* Table S3 for further information relative to single detected protein. Gels are representative of three experiments. (**C**) Comparative proteomic profile. Graph represents protein expression levels in β-TC3 (black peaks) and β-TC3R (grey peaks). Relative volumes of the spots (V%) were calculated by ImageMaster 2D Platinum software, the same software used for gel analysis. (**D**) Proteomic groups. Proteins characterized by significant variation are grouped into four different classes. (**E**) Proteomic network. Protein interaction network reveals that 28 out of 99 proteins are differentially expressed, which are distributed into groups of different biological functions (mainly belonging to cell death pathway, negative regulators of apoptosis and protein folding).

Proteomic technique has been widely used to investigate the protein changes elicited by exposure of islets or insulinoma cells to glucose [21], [22], cytokines [23], [24] and others substances [25], [26]. Here we report the proteomic characterization of β-TC3R. Each maps, performed in triplicate experiments, showed qualitative and quantitative differences of protein profiles in β-TC3 (Fig. 5A) *vs* β-TC3R cells (Fig. 5B). The collection of proteins identified contained 190 proteins in β-TC3R cells (Table S3): among those, 99 proteins (52.1%), including isoforms, were differentially expressed (Fig. 5C). In this last group, 83 were upregulated (43.7%) and 16 were downregulated (8.4%). The majority of upregulated proteins in β-TC3R cells included negative apoptosis regulators (ANXA5, GSTP1, HSP and GRP proteins), galactic protein family member (LEG1), stress response proteins (SUMO4, NPM, PRDX2c, NDKA, NDKB, FKB1, PPIF, TCPE, Terabit, PSB5, DDAH1), glycolytic enzymes (ALDOA, ENOA, FUMH, G3P, IPYR, KPYM, LDHA, PGK, PNPH, TPIS, PGK and LDHB), redox protein family members (PRDX1, PRDX4, SODC), cell-cycle and biosynthesis regulators (PDIA3 members), specific components of the catabolic protein machinery (UBIQ, UCHL1), calcium-binding protein family (S10A6, S10A4, S10AB), protein binding (ACBP and C1QBP) and cytoskeleton (PROF1, VINC a and b, IF6) (Fig. 5D).

The predictive protein interaction analysis showed the complex network of proteins involved in cell death, inhibition of apoptosis and folding (Fig. 5E), suggesting the existence of protein cross-talk. Acronyms and details on identified proteins are listed in Table S3.

### SUMO4 expression, NF-*k*B and JAK/STAT signalling pathways in β-TC3R cells

**Figure 6:**
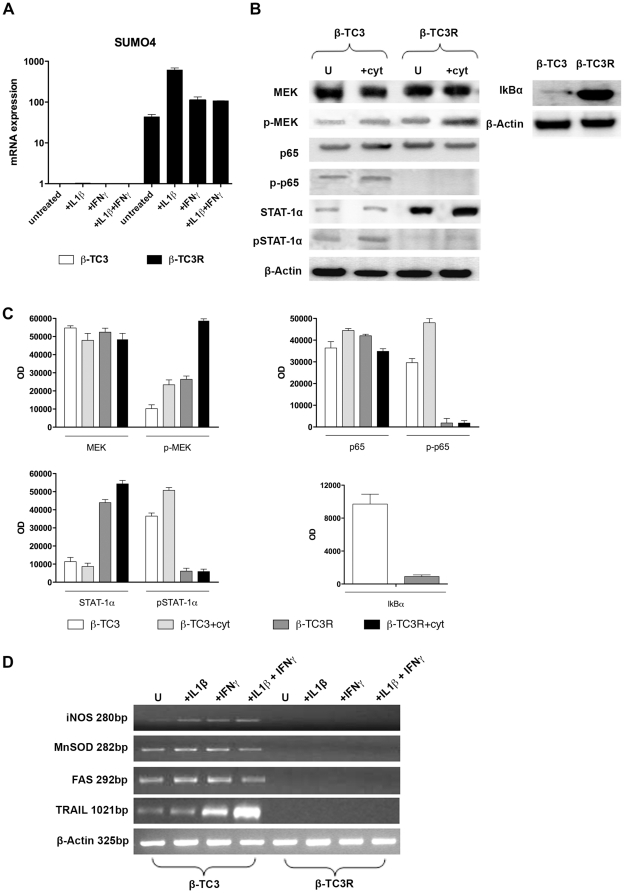
SUMO4 hyperexpression blocks NF-*k*B and JAK/STAT signalling pathways in β-TC3R cells. (**A**) Analysis of SUMO4 gene expression by qRT-PCR in β-TC3 and β-TC3R cells after cytokines exposure compared with untreated cells. SUMO4 is undetectable in β-TC3 cells, while it is strongly upregulated after cytokine exposure in β-TC3R cells. Cytokine treatment is 100 IU/ml IL-1β, 100 IU/ml IFN-γ or their combination, for 72 hours. (**B**) Effects of cytokine treatment on unphosphorilated and phosphorylated forms of Mek, p65 and Stat-1α. Cytokine treatment upregulates p-Mek protein levels in β-TC3R cells in comparison to β-TC3; whereas it does not activate p-65 and Stat-1α. U: untreated; Cyt: 100 IU/ml IL-1β + IFN-γ for 20 min. Data shown are representative of 5 independent experiments. (**C**) Quantification of proteic bands performed by densitometric analysis. OD: optical density units. Each bar represents average ± SD of 5 independent experiments. (**D**) NF-*k*B and JAK/STAT signalling pathways are blocked in β-TC3R cells. Cytokine treatment induces expression of NF-*k*B and the JAK/STAT-dependent genes iNOS, MnSOD, FAS and TRAIL, in β-TC3 cells compared to untreated cells. By contrast, these genes are not expressed in both untreated and cytokine-treated β-TC3R cells. U: untreated; cytokine treatment is 100 IU/ml IL-1β, 100 IU/ml IFN-γ or their combination, after 72 hours. bp = base pairs.

Proteomic analysis of β-TC3R cells showed upregulation of SUMO4, a stress responses protein acting as negative feedback regulator in NF-*k*B and JAK/STAT signalling pathways. This result was confirmed by qRT-PCR, which showed a remarkable increase in SUMO4 expression in untreated β-TC3R cells compared to untreated β-TC3 cells (50±5.9 vs 0.015±0.001, respectively; p<0.005). In addition, IL-1β treatment for 72 hours in β-TC3R cells determined a 14-fold increase compared to their respective untreated cells (Fig. 6A).

Western blot analysis (Fig. 6B) revealed that after 72-hour cytokine treatment, phosporilated Mek (p-Mek) was higher in β-TC3R than in β-TC3 cells (48.56±0.2 *vs* 25.70±0.8, respectively; p<0.005), as expression of higher proliferation rate. In addition, both phosphorylated p65 (p-p65) and STAT-1α (pSTAT-1α were were undetectable in β-TC3R compared to sensitive β-TC3 cells. Notably, the lack of pSTAT-1α by accumulation of the unphosphorilated form STAT-1α, as well as the NF-*k*B inhibitory protein I*k*Bα (β-TC3R compared to β-TC3: 189.71±0.3 *vs* 47±0.2 and 176.03±0.2 vs 2.21±0.3, respectively; p<0.005). Quantification of western blot bands by optical density analysis is shown in Fig. 6C. RT-PCR analysis further confirmed the inhibition of the NF-*k*B and JAK/STAT signalling pathways, as iNOS, MnSOD and FAS/CD95 (which are NF-*k*B-induced genes) and TRAIL (a STAT-induced gene) mRNA expression resulted absent in β-TC3R cells (Fig. 6C).

### Silencing of SUMO4 restores cytokine sensitivity in β-TC3R

**Figure 7:**
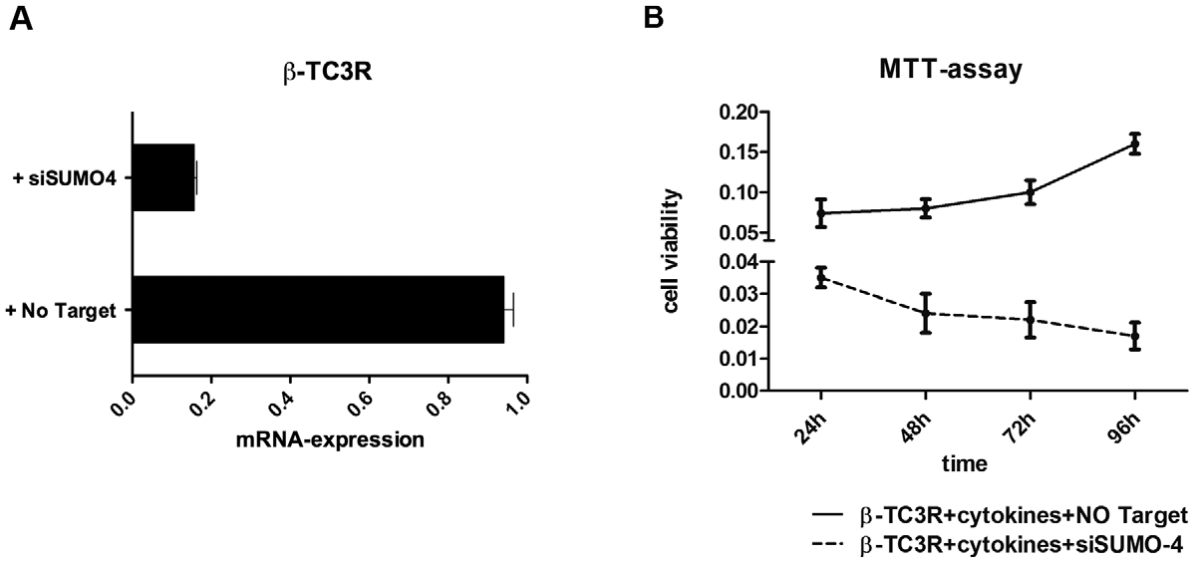
Silencing of SUMO4 restores cytokine sensitivity in β-TC3R. (**A**) Efficacy of SUMO4 silencing after 72 hours assessed by qRT-PCR analysis. (**B**) Loss of cytokine-resistance after SUMO4 silencing. MTT assay shows reduction of cell viability in cytokine treated β-TC3R cells after silencing with SUMO4 siRNA compared to non-targeting siRNA (47.3%, 30%, 22%, 10.6% after 24, 48, 72 and 96 h, respectively).

To verify the relationship between SUMO4 and the acquired cytokine resistance of β-TC3R cells, we silenced SUMO4 using a specific small interfering RNA (siRNA). Silencing efficacy (73%) is shown in Fig. 7A. As demonstrated by MTT assay, siRNA treatment caused loss of cytokine resistance in β-TC3R cells. The reduction of cell viability in cells treated with siRNA compared to non-targeting was 47.3%, 30%, 22%, and 10.6% after 24, 48, 72 and 96 hours, respectively (Fig. 7B).

## Discussion

In our study we aimed at investigating the possible mechanisms involved in resistance to cytokine-induced β-cell death. To this purpose, we created a cytokine-resistant β-cell line (β-TC3R) by chronically treating with IL-1β + IFN-γ the cytokine-sensitive murine insulinoma β-TC3 cell line. We found that β-TC3R cells maintained 100% viability after exposure with IL-1β + IFN-γ, showing no increase in Caspase 3 activity, DNA fragmentation or significant elevation of the subG0 cell population. This was accompanied by a much higher proliferation rate in comparison to the parental line. Despite the acquisition of the new cytokine-resistant phenotype, β-TC3R cells maintained the expression of several specific β-cell markers, among them the two nuclear transcriptions factors PDX-1 and NKX6.1, insulin and GLUT2. In addition, the evaluation of the secretory ability showed that β-TC3R cells are able to respond to different stimuli in a regulated manner by secreting mature insulin (assessed as C-peptide), although at a lower levels in comparison to cytokine-sensitive β-TC3 cells. In particular, while the incubation with KCl and tolbutamide caused only mildly reduced secretion levels in β-TC3R cells, the glucose-induced response resulted consistently impaired. This difference is likely due to a cytokine-mediated modification in the expression of the genes responsible for glucose-induced insulin secretion, such as GLUT2 and glucokinase. However, both β-TC3 and β-TC3R cells have comparable expression of GLUT2 in immunofluorescence, suggesting that it cannot account for the impaired glucose-induced insulin secretion found in β-TC3R cells. By contrast, glucokinase expression was severely reduced in β-TC3R cells by western blot analysis. Glucokinase is a rate-limiting enzyme in β-cell glycolysis and is thought to be the intracellular sensor for glucose-induced insulin secretion, because it has a *K*_m_ higher than the physiological concentration of glucose [27], [28]. Therefore a downregulation of glucokinase would be compatible with the impaired response observed in cytokine-resistant cells. This is further supported by the only moderately reduced c-peptide secretion observed after incubation with KCl and tolbutamide, as the continuous and strong membrane depolarization induced by these two stimuli bypasses the intracellular “glucose sensor”.

Several possible mechanisms involved in the acquisition of cytokine resistance in β-TC3R were investigated. First, we found downregulation of IL-1RI and IFN-γR. However, the decreased IL-1R1 and IFN-γR expression in β-TC3R cells is not sufficient *per se* to explain cytokine resistance, as cells may increase receptor binding affinity as compensatory mechanism [19]. Interestingly, we also found that β-TC3R cells express higher levels of SOCS-3 and SUMO4 proteins, in addition to the inactivation of NF-*k*B and STAT-1α pathways. SOCS-3 is a classical negative regulator of cytokine signalling, which has been shown to act as a negative feedback regulator of IL-1β, preventing its action on gene expression profile induced by STAT-1α, NF-*k*B and MAPK [1]–[3], [6], [29], [30]. Taken together, these findings could be responsible for the acquired cytokine resistance.

A more large-scale analysis of protein expression pattern was obtained by proteomic analysis. We identified 99 differentially expressed protein spots (including isoforms); among these, 83 were upregulated and 16 were downregulated in β-TC3R cells. Many upregulated proteins belonged to families with either metabolic or chaperone functions. In particular, in β-TC3R cells we found upregulation of many proteins involved in glycolysis and Krebs cycle (ALDOA, ENOA, FUMH, G3P, IPYR, KPYM, LDHA, PGK, PNPH, TPIS, PGK and LDHB), cell cycle and biosynthesis regulators (PDIA3 members which catalyze SS bond rearrangements and the master regulator of the unfolded protein GRP78). These findings are consistent with the higher growth rate exhibited by β-TC3R cells in comparison to sensitive β-TC3 cells [31], [32]. At the same time, negative regulators of apoptosis and stress responses proteins (chaperones, heath shock proteins, oxidoreductases) were found increased in β-TC3R cells, suggesting that this pattern is due to an adaptation to the continuous cytokine exposure. In particular, among proteins belonging to the latter category, we found upregulation of DDAH1 and PSB5. Altered DDAH1 protein expression suggests increased nitric oxide production and increased oxidative stress [33]. PSB5 plays a role in the protection against oxidative damage through the Nrf2/ARE pathway. Activation of Nrf2/ARE pathway protects endothelial cells from oxidant injury and inhibits inflammatory gene expression [34]. Furthermore, members of redox protein family (PRDX1, PRDX4, SODC) were found upregulated. The observed induction of SODC in beta cells after cytokines exposure is consistent with previous findings obtained with microarray or Western Blot analysis [35]. A long exposure of β-cells to cytokines results in upregulation of β-cell ROS scavenging machinery. Thus, our resistant cells seem to be characterized by several protective mechanisms against oxidative stress and apoptotic cell death. In our study we also describe proteins not previously identified by proteomic analysis, such as catabolic machinery members (UBIQ, UCHL1), and calcium-binding proteins family (S10A6, S10A4, S10AB). S10A proteins, involved in the reorganization of the actin cytoskeleton, could explain the different morphology of β-TC3R in comparison to β-TC3 cells.

SUMO4 was the only protein implicated in cytokine signalling that was found upregulated by proteomic analysis. Recent studies suggested a role for SUMO4 in prevention of apoptosis by specific targeted SUMOylation of anti-oxidant enzymes, chaperones, DNA damage signalling and anti-stress transcription factors [36], [37]. SUMO4 also plays a role as a negative feedback regulator in NF-*k*B and JAK/STAT signalling pathways [38]. Upon cytokine stimulation, NF-*k*B signalling initiates a kinase cascade, which eventually activates I*k*Bα kinase (IKK) complex to phosphorylate I*k*Bα and dissociate it from SUMO4 conjugation. Dissociated I*k*Bα is thereafter rapidly degraded by the ubiquitin/proteasome pathway, then NF-*k*B is activated and translocated into the nucleus where it binds to promoters, and activates gene transcription [39], [40]. Thus, the remarkable upregulation of SUMO4 found in β-TC3R cells could explain the accumulation of I*k*Bα in the cytosol and the consequent absence of p65 phosphorylation. In particular, SUMO4 expression rapidly increased upon IL-1βexposure. These findings suggest the NF-*k*B signalling is blocked or severely impaired, as confirmed by the absence of expression of its downstream genes, i.e. iNOS (inducible nitric oxide synthase) [30], MnSOD (Manganese superoxide dismutase, a free radical scavenger) and FAS (CD95) [41], [42].

Interestingly, we found simultaneous STAT-1α hyperexpression and lack of its phosphorylated form pSTAT-1α, as well as absence of TRAIL (Tumor Necrosis Factor-alpha-related apoptosis-inducing ligand), which represents its target gene. This phenomenon could be due to SUMOylation of STAT-1α, which it is known to inhibit its DNA-binding activity. Our findings are consistent with Chen et al. who described an increased expression of total STAT-1α protein in long-term cell cultures in the presence of cytokines [43]. In addition, recent studies have showed that a genetic susceptibility of SUMO4 may be implicated in the pathogenesis of T1DM. Sequence analysis of diabetic patients and controls revealed that a mutation (M55V) located in one of the SUMO4 domains results in impaired SUMO4, leading to higher levels of activated NF-*k*B, which in turn transcribes immune response genes [44], [45]. In our opinion, hyperespression of SUMO4 could be the key event responsible for cytokine resistance in β-TC3R cells by blocking JAK/STAT and NF-*k*B lethal signalling. To confirm this hypothesis, we silenced SUMO4 in β-TC3R cells. Silencing resulted in a significant reduction of cell viability after cytokine treatment. The high silencing rate (73%) correlates with the increased killing activity of IL-1β and IFN-γ up to 89%, confirming direct SUMO4 effect on cytotoxicity. So far, no similar effect in preventing lethal effect of cytokines was ever found after inhibition of a single factor.

In conclusion, our study represents the first extensive proteomic characterization of a murine cytokine-resistant β-cell line. This knowledge might be useful to investigate the different pathways involved in the development of cytokine resistance and define new strategies for preservation of the β-cell mass during immune-mediated inflammation. In addition, unraveling the mechanisms behind β-cell cytokine resistance may be also exploited for prolonging the survival of transplanted islets or engineered β-cells in Type 1 diabetes.

## Supporting Information

Table S1 **Antibodies used for immunocytochemical staining and Western Blot detection.**
(DOC)

Table S2 **Primer pair sequences, cDNA fragment sizes and annealing temperatures used for RT-PCR.**
(DOC)

Table S3 **Identification of protein expression levels in β-TC3R cells versus β-TC3 cells.**
(PDF)
