# Supplementary material for: In Vitro Phenotypic, Genomic and Proteomic Characterization of a Cytokine-Resistant Murine β-TC3 Cell Line
Source: PLoS One. 2012 Feb 29;7(2):e32109. doi: 10.1371/journal.pone.0032109 (PMC3290556; doi:10.1371/journal.pone.0032109)
Supplement: Table S1 — Antibodies used for immunocytochemical staining and Western Blot detection. (DOC) [file pone.0032109.s001.doc]

Table S1:

| **ANTIBODY** | **HOST** | **CODE NUMBER** | **DILUTION** |
| --- | --- | --- | --- |
| GLUT2 | Rabbit | Santa Cruz, sc-9117 | 1:50 |
| Insulin | Guinea pig | Dako, A0564 | 1:100 |
| PDX1 | Rabbit | Millipore, AB3503 | 1:100 |
| Nkx6.1 | Mouse | Gift from Ole Madsen | 1:500 |
| SOCS3 | Mouse | Abcam, ab78341 | 1:1000 |
| MEK-1/2 | Rabbit | Abcam, ab70613 | 1:500 |
| p-MEK-1/2 Ser218-222 | Rabbit | Abcam, ab78132 | 1:500 |
| IkB-α | Rabbit | Abcam, ab32518 | 1:1000 |
| p65 | Rabbit | Santa Cruz, (C20) sc-372 | 1:500 |
| Phospho-p65 Ser536 | Rabbit | Santa Cruz, sc-33020 | 1:500 |
| STAT1-α | Rabbit | Santa Cruz, sc-591 | 1:500 |
| p-STAT1-α Ser727 | Rabbit | Santa Cruz, sc-16570 | 1:500 |
| GCK (H-88) | Rabbit | Santa Cruz, sc-7908 | 1:500 |
