# Supplementary material for: In Vitro Phenotypic, Genomic and Proteomic Characterization of a Cytokine-Resistant Murine β-TC3 Cell Line
Source: PLoS One. 2012 Feb 29;7(2):e32109. doi: 10.1371/journal.pone.0032109 (PMC3290556; doi:10.1371/journal.pone.0032109)
Supplement: Table S2 — Primer pair sequences, cDNA fragment sizes and annealing temperatures used for RT-PCR. (DOC) [file pone.0032109.s002.doc]

Table S2:

| **NAME** | **Sequence** | **bp** | **Melting Temperature** |
| --- | --- | --- | --- |
| iNOS | 5'-GACAGCACAGAATGTTCCAG-3'  5'-TGGCCAGATGTTCCTCTATT-3' | 280 | 55ºC |
| MnSOD | 5'-GACCTGCCTTACGACTATGG-3'  5'-GACCTTGCTCCTTATTGAAGC-3' | 282 | 55ºC |
| Fas | 5'-TGCACCTCGTGTGGACTTGA-3'  5'-GGAACTTTGTTTCTTGCATT-3' | 292 | 56ºC |
| Trail | 5'-AAGACCTTAGGCCAGAAGAT-3'  5'-CTCTGCAAACTGATACCAGG-3' | 1021 | 55ºC |
| β-Actin | 5’-TGACGGGGTCACCCACACTGTGCCCATCTA-3'  5’-CTAGAAGCACTTGCGGTGGACGATGGAGGG-3' | 325 | 57°C |
| SOCS-3 | 5'-ATGGTCACCCACAGCAAGTT-3'  3'-AATCCGCTGTCCTGCAGCTT-5' | 69 | 60°C |
| SUMO4 | 5'-AGGTGGCGGGACAGGATGGT-3' 5'-TGCTTCACTGACAATCCCCGTG-3' | 55 | 60°C |
| IL-1βRI | 5'-TCGCCACTGGGGCACCCGGGC-3' 5'-CCAGCGACAGCAGAGGCACC-3’ | 87 | 60°C |
| IFN-γRI | 5'-CCTTGTAGCCTCACCGCCTA-3' 5'-TGGAGGGTCGTGCTCTGCCA-3' | 136 | 60°C |
