# Supplementary material for: In Vitro Phenotypic, Genomic and Proteomic Characterization of a Cytokine-Resistant Murine β-TC3 Cell Line
Source: PLoS One. 2012 Feb 29;7(2):e32109. doi: 10.1371/journal.pone.0032109 (PMC3290556; doi:10.1371/journal.pone.0032109)
Supplement: Table S3 — Identification of protein expression levels in β-TC3R cells versus β-TC3 cells. (PDF) [file pone.0032109.s003.pdf]

Protein name	AC	Abbreviated Name	Theoretical MW	Exp.MW	Theoretical Pi	Exp. Pi	ID methods	% MASSES MATHCHED	Sequence coverage (%) N-terminal residues	SPECIES
14-3-3 protein gamma	P61981	1433G	28302	29255	4,8	4,84	1	78	41	Human homolog
14-3-3 protein zeta/delta	P63101	1433Z	27771	16471	4,73	5,52	1	26	41	Mouse
Acyl-CoA-binding protein	P07108	ACBP	10044	9951	6,12	5,95	1	80	50	Human homolog
Aconitate hydratase, mitochondrial	Q99798	ACON	85425	82624	7,36	6,97	1	58	22	Human homolog
Actin, cytoplasmic 1	P60709	ACTB/G	41737	42000	5,29	5,30	1	86	19	Human homolog
Actin, cytoplasmic 1	P60709	ACTB/G fr a	41737	24561	5,29	5,28	1	21	20	Human homolog
Actin, cytoplasmic 1	P60709	ACTB/G fr b	41737	21734	5,29	5,09	1	19	35	Human homolog
Actin, cytoplasmic 1	P60709	ACTB/G fr c	41737	37690	5,29	5,56	1	21	34	Human homolog
Actin, cytoplasmic 1	P60710	ACTB/G fr d	41737	36863	5,29	5,55	1	16	22	Mouse
Actin, cytoplasmic 1	P60710	ACTB/G fr e	41737	31616	5,29	5,58	1	19	15	Mouse
Actin, cytoplasmic 1	P60709	ACTB/G fr f	41737	30412	5,29	5,57	1	50	13	Human homolog
Aldo-keto reductase family 1 member A1	P14550	AK1A1	36573	38698	6,32	6,62	1	35	28	Human homolog
Aldo-keto reductase family 1 member B10	O60218	AK1BA	36021	36506	7,12	6,99	1	38	36	Human homolog
Fructose-bisphosphate aldolase A	P04075	ALDOA a	39420	37171	8,3	7,30	1	62	59	Human homolog
Fructose-bisphosphate aldolase A	P05064	ALDOA b	39356	37171	8,3	7,38	1	13	31	Mouse
Acidic leucine-rich nuclear phosphoprotein 32 family member A	P39687	AN32A	28585	31054	3,99	4,03	1	31	30	Human homolog
Annexin A1	P04083	ANXA1 a	38714	36659	6,57	6,52	1	30	44	Human homolog
Annexin A1	P04083	ANXA1 b	38714	37586	6,57	6,78	1	33	27	Human homolog
Annexin A2	P07355	ANXA2 a	38604	37171	7,57	6,91	1	46	29	Human homolog
Annexin A2	P07356	ANXA2 b	38676	37274	7,55	7,08	1	22	35	Mouse
Annexin A5	P48036	ANXA5	35752	34269	4,83	5,01	1	31	23	Mouse
Adenine phosphoribosyltransferase	P07741	APT	19608	17653	5,78	5,56	1	23	36	Human homolog
Actin-related protein 2/3 complex subunit 5-like protein	Q9D898	ARP5L	16980	16123	6,31	6,23	1	100	60	Mouse
Actin-related protein 2/3 complex subunit 5	O15511	ARPC5	16320	15205	5,47	5,67	1	69	48	Human homolog
ATP synthase subunit alpha, mitochondrial	P25705	ATPA	59751	49695	9,16	7,23	1	26	38	Human homolog
ATP synthase subunit beta, mitochondrial	P06576	ATPB	56560	49878	5,26	5,16	1	89	59	Human homolog
Beta-2-microglobulin	P61769	B2MG	13715	9872	6,06	6,71	2	-	res. 21-30	Human homolog
Complement component 1 Q subcomponent-binding protein, mitochondrial	Q07021	C1QBP	31362	34474	4,74	4,54	1	16	37	Human homolog
Calreticulin	P27797	CALR	48142	61439	4,29	4,52	1	29	35	Human homolog
Cathepsin D	P07339	CATD a	44552	31427	6,1	5,77	1	75	19	Human homolog
Cathepsin D	P07339	CATD b	44552	29342	6,1	6,23	1	59	26	Human homolog
Chloride intracellular channel protein 1	O00299	CLIC1	26923	32187	5,09	5,21	1	48	43	Human homolog
Cofilin-1	P23528	COF1 a	18502	15409	8,22	6,42	1	45	61	Human homolog
Cofilin-1	P23528	COF1 b	18502	14964	8,22	7,22	1	53	42	Human homolog
Cofilin-1	P18760	COF1 c	18502	14187	8,22	7,23	1	83	28	Mouse
Calcyclin-binding protein	Q9CXW3	CYBP	26510	26306	7,64	7,48	1	23	24	Mouse
Cystatin-B	P04080	CYTB	11140	12449	6,96	6,63	1	100	61	Human homolog
N(G),N(G)-dimethylarginine dimethylaminohydrolase 1	O94760	DDAH1	31122	37848	5,53	5,61	1	27	56	Human homolog
N(G),N(G)-dimethylarginine dimethylaminohydrolase 2	O95865	DDAH2	29644	30777	5,66	5,72	1	27	35	Human homolog
Dihydrolipoyl dehydrogenase, mitochondrial	P09622	DLDH	54177	59450	7,95	6,82	1	71	28	Human homolog
Elongation factor 1-beta	P24534	EF1B	24764	32476	4,5	4,66	1	50	29	Human homolog
Elongation factor 2	P58252	EF2	95338	101225	6,41	6,88	1	28	11	Mouse
Elongation factor Tu, mitochondrial	Q8BFR5	EFTU	49542	44858	7,26	6,59	1	21	24	Mouse
Eukaryotic translation initiation factor 3 subunit I	Q9QZD9	EIF3I	36502	38377	5,38	5,57	1	38	34	Mouse
Alpha-enolase	p06733	ENOA a	47169	46359	7,01	6,38	3	-	-	Human homolog
Alpha-enolase	p06733	ENOA b	47169	46190	7,01	6,52	3	-	-	Human homolog
Alpha-enolase	P06733	ENOA c	47169	45519	7,01	6,70	1	50	41	Human homolog
Alpha-enolase	p06733	ENOA d	47169	45187	7,01	6,87	3	-	-	Human homolog
Alpha-enolase	P17182	ENOA fr a	47141	38859	6,37	6,13	1	77	22	Mouse
Alpha-enolase	P06733	ENOA fr b	47169	40287	7,01	6,90	2	-	res. 57-66	Human homolog
S-formylglutathione hydrolase	P10768	ESTD a	31463	33162	6,54	6,53	1	17	25	Human homolog
S-formylglutathione hydrolase	P10768	ESTD b	31463	32573	6,54	6,59	1	21	23	Human homolog
S-formylglutathione hydrolase	P10768	ESTD c	31463	32573	6,54	6,71	1	18	72	Human homolog
Fatty acid-binding protein, epidermal	Q01469	FABP5	15164	12583	6,6	6,33	1	19	57	Human homolog
Fatty acid-binding protein, brain	O15540	FABP7 a	14889	11993	5,4	5,31	1	28	93	Human homolog
Fatty acid-binding protein, brain	O15541	FABP7 b	14889	12089	5,4	5,67	1	100	25	Human homolog
Peptidyl-prolyl cis-trans isomerase FKBP1A	P62942	FKB1A	11951	11462	7,89	7,24	1	64	63	Human homolog
Fumarate hydratase, mitochondrial	P07954	FUMH	54637	43406	8,85	7,01	1	63	21	Human homolog
Glyceraldehyde-3-phosphate dehydrogenase	P04406	G3P a	36053	36506	8,57	7,09	1	73	26	Human homolog
Glyceraldehyde-3-phosphate dehydrogenase	p04406	G3P b	36053	36355	8,57	7,21	2	-	res. 2-11	Human homolog
Glyceraldehyde-3-phosphate dehydrogenase	p04406	G3P c	36053	36254	8,57	7,34	2	-	res. 2-21	Human homolog
Glyceraldehyde-3-phosphate dehydrogenase	p04406	G3P d	36053	36153	8,57	7,40	2	-	res. 2-11	Human homolog
Glyceraldehyde-3-phosphate dehydrogenase	p04406	G3P e	36053	36053	8,57	7,52	2	-	res. 2-31	Human homolog
Neutral alpha-glucosidase AB	Q14697	GANAB	106874	98742	5,74	5,95	1	32	21	Human homolog
Guanine nucleotide-binding protein subunit beta-2-like 1	P63244	GBLP	35077	31710	7,6	6,89	1	20	35	Human homolog
Rho GDP-dissociation inhibitor 1	P52565	GDIR	23207	24338	5,03	5,06	1	23	38	Human homolog
Rho GDP-dissociation inhibitor 2	P52566	GDIS	22988	23047	5,1	5,16	1	28	64	Human homolog
Gelsolin	P06396	GELS	85698	81723	5,9	5,91	1	100	40	Human homolog
Glucosidase 2 subunit beta	P14314	GLU2B	59425	82927	4,33	4,58	1	24	20	Human homolog
75 kDa glucose-regulated protein	P38646	GRP75 a	73680	70342	5,87	5,61	1	82	24	Human homolog
75 kDa glucose-regulated protein	P38647	GRP75 b	73528	65861	5,91	5,88	1	37	37	Mouse
78 kDa glucose-regulated protein	P11021	GRP78	72333	73498	5,07	5,11	1	66	49	Human homolog
94 kDa glucose-regulated protein	P14625	ENPL	92469	91200	4,76	4,90	1	50	30	Human homolog
Glutathione S-transferase omega-1	P78417	GSTO1 a	27566	31147	6,24	5,91	1	19	27	Human homolog
Glutathione transferase omega-1	P78417	GSTO1 b	27566	30777	6,24	6,22	1	18	60	Human homolog
Glutathione S-transferase P	P09211	GSTP1 a	23356	23356	5,43	5,36	1	71	34	Human homolog
Glutathione S-transferase P	P19157	GSTP1 b	23609	23356	7,69	5,64	1	29	52	Mouse
Heterogeneous nuclear ribonucleoprotein H	O35737	HNRH1	49199	50060	5,89	6,33	1	91	23	Mouse
Heat shock protein beta-1	P04792	HSP27 a	22783	27314	5,98	6,09	1	31	52	Human homolog
Heat shock protein beta-1	P04792	HSP27 b	22783	26306	5,98	5,79	1	27	29	Human homolog
Heat shock protein beta-1	P04792	HSP27 c	22783	26547	5,98	6,07	1	52	61	Human homolog
60 kDa heat shock protein, mitochondrial	P10809	HSP60 a	61055	61215	5,7	5,28	2	-	res. 33-38	Human homolog
60 kDa heat shock protein, mitochondrial	P10809	HSP60 b	61055	60547	5,7	5,35	1	78	39	Human homolog
Heat shock 70 kDa protein 1	P08107	HSP71	70052	67568	5,48	5,63	1	38	38	Human homolog
Heat shock 70 kDa protein 4	P34932	HSP74	94331	103770	5,11	5,26	1	58	18	Human homolog
Heat shock cognate 71 kDa protein	P11142	HSP7C a	70898	-	5,37	-	1	22	21	Human homolog
Heat shock cognate 71 kDa protein	P11142	HSP7C b	70898	68816	5,37	5,37	1	23	28	Human homolog
Heat shock cognate 71 kDa protein	P11142	HSP7C c	70898	68564	5,37	5,43	1	70	43	Human homolog
Heat shock cognate 71 kDa protein	P11142	HSP7C d	70898	68065	5,37	5,49	1	38	38	Human homolog
Heat shock cognate 71 kDa protein	P63017	HSP7C fr	70871	14964	5,37	6,47	1	31	11	Mouse
Eukaryotic translation initiation factor 5A-1	P63242	IF5A1	16832	14964	5,07	5,17	1	23	35	Mouse
Eukaryotic translation initiation factor 6	O55135	IF6	26511	28057	4,63	4,68	1	80	43	Mouse
Inorganic pyrophosphatase	Q9D819	IPYR	32667	37068	5,37	5,65	1	29	23	Mouse
Thiosulfate sulfurtransferase/rhodanese-like domain-containing protein 1	Q8NFU3	TSTD1	12530	10552	5,85	5,61	1	100	31	Human homolog
Pyruvate kinase isozymes M1/M2	P14618	KPYM a	57937	58801	7,96	7,07	1	25	39	Human homolog

Pyruvate kinase isozymes M1/M2	P14618	KPYM b	57937	59233	7,96	7,14	1	89	53	Human homolog
L-lactate dehydrogenase A chain	P00338	LDHA	36689	34681	8,44	7,30	1	50	17	Human homolog
L-lactate dehydrogenase B chain	P16125	LDHB	36572	37171	5,7	5,82	1	63	14	Mouse
Galectin-1	P16045	LEG1	14866	11709	5,32	5,10	1	86	27	Mouse
Galectin-3	P17931	LEG3	26152	27314	8,58	7,44	1	47	26	Human homolog
Lactoylglutathione lyase	Q04760	LGUL	20778	19021	5,12	5,04	1	56	22	Human homolog
Lamin-A/C	P02545	LMNA	74139	44045	6,57	5,99	1	22	19	Human homolog
Macrophage migration inhibitory factor	P14174	MIF a	12476	10031	7,73	7,07	1	100	23	Human homolog
Macrophage Migration Inhibitory Factor	P14174	MIF b	12476	10220	7,73	7,16	1	80	21	Human homolog
Nucleoside diphosphate kinase A	P15532	NDKA	17208	18130	6,84	5,95	1	42	43	Mouse
Nucleoside diphosphate kinase B	Q01768	NDKB	17363	15084	6,97	7,44	1	25	40	Mouse
Nucleophosmin	Q61937	NPM	32560	17189	4,62	4,90	1	25	33	Mouse
Nuclear transport factor 2	P61970	NTF2	14478	10637	5,1	4,94	1	37	53	Human homolog
Proliferation-associated protein 2G4	Q9UQ80	PA2G4	43787	43565	6,13	6,42	1	67	16	Human homolog
Protein DJ-1	Q99LX0	PARK7	20021	21561	6,32	6,26	1	24	34	Mouse
Protein disulfide-isomerase	P07237	PDIA1	57116	58801	4,76	4,86	1	33	9	Human homolog
Protein disulfide-isomerase A3	P30101	PDIA3 a	56782	54454	5,98	5,74	1	57	21	Human homolog
Protein disulfide-isomerase A3	P30101	PDIA3 b	56782	54057	5,98	5,86	1	57	27	Human homolog
Protein disulfide-isomerase A3	P27773	PDIA3 c	56678	54255	5,88	5,99	1	29	24	Mouse
Protein disulfide-isomerase A3	P30101	PDIA3 d	56782	54454	5,98	6,12	1	33	49	Human homolog
Phosphatidylethanolamine-binding protein 1	P30086	PEBP1	21057	17419	7,01	7,08	1	33	52	Human homolog
Prefoldin subunit 2	O70591	PFD2	16534	14300	6,2	6,24	1	64	40	Mouse
Phosphoglycerate mutase 1	P18669	PGAM1 a	28804	28225	6,67	6,60	1	39	51	Human homolog
Phosphoglycerate mutase 1	P18669	PGAM1 b	28804	28225	6,67	6,78	1	14	59	Human homolog
Phosphoglycerate kinase 1	P00558	PGK 1 a	44615	41364	8,3	7,05	1	26	17	Human homolog
Phosphoglycerate kinase 1	P00558	PGK 1 b	44615	41192	8,3	7,20	1	26	17	Human homolog
Phosphoglycerate kinase 1	P09411	PGK1 c	44550	41021	8,02	7,32	1	43	28	Mouse
Phosphoglycerate kinase 1	P00558	PGK1 d	44615	40964	8,3	7,39	1	67	13	Human homolog
Prohibitin	P67778	PHB	29820	28650	5,57	5,61	1	35	68	Mouse
Purine nucleoside phosphorylase	P00491	PNPH a	32118	30231	6,45	6,53	1	60	50	Human homolog
Purine nucleoside phosphorylase	P00491	PNPH b	32118	28479	6,45	6,47	1	24	66	Human homolog
Pyridoxine-5'-phosphate oxidase	Q9NVS9	PNPO	29988	27974	6,61	6,21	1	33	27	Human homolog
Peptidyl-prolyl cis-trans isomerase A	P62937	PPIA a	18012	15124	7,68	6,93	1	20	37	Human homolog
Peptidyl-prolyl cis-trans isomerase A	P62937	PPIA b	18012	14300	7,68	6,71	1	64	50	Human homolog
Peptidyl-prolyl cis-trans isomerase A	P62937	PPIA c	18012	14224	7,68	6,94	1	43	45	Human homolog
Peptidyl-prolyl cis-trans isomerase A	P62937	PPIA d	18012	14339	7,68	7,00	1	73	65	Human homolog
Peptidyl-prolyl cis-trans isomerase A	P62937	PPIA e	18012	14454	7,68	7,13	1	33	51	Human homolog
Peptidyl-prolyl cis-trans isomerase B	P23284	PPIB	23743	16471	9,42	7,79	1	85	49	Human homolog
Peptidyl-prolyl cis-trans isomerase F, mitochondrial	Q99KR7	PPIF	21737	16692	9,32	7,48	1	33	14	Mouse
Peroxiredoxin-1	P35700	PRDX1 a	22176	20550	8,26	6,44	1	100	57	Mouse
Peroxiredoxin-1	Q06830	PRDX1 b	22110	20938	8,27	6,78	1	11	73	Human homolog
Peroxiredoxin-1	Q06830	PRDX1 c	22110	21792	8,27	7,05	1	63	45	Human homolog
Peroxiredoxin-1	Q06830	PRDX1 d	22110	21908	8,27	7,27	1	73	55	Human homolog
Peroxiredoxin-2	P32119	PRDX2	21892	20010	5,66	5,63	1	17	78	Human homolog
Peroxiredoxin-3	P30048	PRDX3	27693	23356	7,68	6,14	1	22	64	Human homolog
Peroxiredoxin-4	Q13162	PRDX4	30540	27314	5,86	5,75	1	9	57	Human homolog
Profilin-1	P07737	PROF1 a	15054	11615	8,44	6,95	1	71	40	Human homolog
Profilin-1	P62962	PROF1 b	14957	11678	8,46	7,28	1	43	50	Mouse
Profilin-1	P07737	PROF1 c	15054	11898	8,44	7,51	1	78	41	Human homolog
Pre-mRNA-processing factor 19	Q99KP6	PRP19	55239	38805	6,14	6,38	1	100	12	Mouse
26S protease regulatory subunit 8	P62195	PRS8	45626	43565	7,11	6,94	1	63	15	Human homolog
Proteasome subunit alpha type-5	p28066	PSA5	26411	28057	4,74	4,77	2	-	res. 4-13	Human homolog
Proteasome subunit alpha type-6	Q9QUM9	PSA6	27372	27559	6,35	6,48	1	19	34	Mouse
Proteasome subunit beta type-3	P49720	PSB3	22949	23896	6,14	6,29	1	100	34	Human homolog
Proteasome subunit beta type-5	O55234	PSB5	28532	17938	6,52	7,44	1	88	36	Mouse
Proteasome activator complex subunit 1	Q06323	PSME1	28723	30322	5,78	5,83	1	33	31	Human homolog
60S acidic ribosomal protein P0	P14869	RLA0	34216	36914	5,91	6,34	1	19	29	Mouse
Heterogeneous nuclear ribonucleoproteins A2/B1	P22626	ROA2	37430	36965	8,97	7,69	1	56	34	Human homolog
Protein S100-A2	P29034	S10A2	11117	9664	4,68	4,64	1	89	38	Human homolog
Protein S100-A4	P26447	S10A4	11729	9978	5,85	5,58	1	100	27	Human homolog
Protein S100-A6	P06703	S10A6 a	10180	8992	5,32	4,96	1	100	28	Human homolog
Protein S100-A6	P14069	S10A6 b	10051	8945	5,3	5,12	1	67	28	Mouse
Protein S100-A11	P31949	S10AB a	11740	10413	6,56	5,36	1	100	53	Human homolog
Protein S100-A11	P50543	S10AB b	11083	10220	5,28	6,10	1	63	34	Mouse
Protein S100-A13	Q99584	S10AD	11471	11012	5,9	5,45	1	68	89	Human homolog
SH3 domain-binding glutamic acid-rich-like protein	O75368	SH3L1	12774	11523	5,22	5,31	2	-	res. 2-11	Human homolog
Superoxide dismutase [Cu-Zn]	P08228	SODC	15943	16383	6,02	5,80	1	100	23	Mouse
Superoxide dismutase [Mn], mitochondrial	P04179	SODM	24722	21503	8,35	6,90	1	20	38	Human homolog
Small ubiquitin-related modifier 4	Q6EEV6	SUMO4	10685	9260	6,57	6,33	1	36	35	Human homolog
Transgelin-2	P37802	TAGL2 sf	22391	12718	8,41	5,69	2	-	res. 12-26	Human homolog
Tubulin alpha-1 chain	P68366	TBA1 a	49924	55256	4,95	5,10	1	57	51	Human homolog
Tubulin alpha-1 chain	P68367	TBA1 b	49924	54653	4,95	5,16	1	76	52	Human homolog
Tubulin alpha-1 chain	P68368	TBA1 c	49924	54454	4,95	5,22	1	57	30	Human homolog
Tubulin beta chain	P07437	TBB5	49671	51547	4,78	5,00	1	52	58	Human homolog
Tubulin beta-5 chain	P99024	TBB5 sf	49671	38217	4,78	5,09	1	43	22	Mouse
T-complex protein 1 subunit epsilon	P80316	TCPE	59624	59886	5,72	5,86	1	24	23	Mouse
Transitional endoplasmic reticulum ATPase	P55072	TERA a	89322	89875	5,14	5,28	1	67	40	Human homolog
Transitional endoplasmic reticulum ATPase	P55072	TERA b	89322	86648	5,14	5,47	1	26	46	Human homolog
Thioredoxin	P10599	THIO a	11737	11370	4,82	4,85	1	100	72	Human homolog
Thioredoxin	P10599	THIO b	11737	11370	4,82	4,97	1	100	66	Human homolog
Triosephosphate isomerase	P60174	TPIS a	26669	26829	6,45	6,35	1	22	42	Human homolog
Triosephosphate isomerase	P60174	TPIS b	26669	26749	6,45	6,59	1	21	41	Human homolog
Triosephosphate isomerase	P60174	TPIS c	26669	26749	6,45	6,71	1	15	28	Human homolog
Triosephosphate isomerase	P60174	TPIS d	26669	26669	6,45	6,81	1	21	52	Human homolog
Tropomyosin beta chain	P58774	TPM2	32837	36355	4,66	4,74	1	61	20	Mouse
Tropomyosin alpha 3 chain	P21107	TPM3	32863	32867	4,68	4,79	1	31	21	Mouse
Transthyretin	P02766	TTHY	15887	13740	5,52	5,60	2	-	res. 21-27	Human homolog
Ubiquitin-conjugating enzyme E2 N	P61088	UBE2N	17138	13486	6,13	6,02	1	89	51	Human homolog
Polyubiquitin-B	P0CG47	UBB	25762	8594	6,86	6,85	2	-	res. 1-15	Human homolog
Ubiquitin carboxyl-terminal hydrolase isozyme L1	P09936	UCHL1	24824	26669	5,33	5,49	1	71	67	Human homolog
Voltage-dependent anion-selective channel protein 1	Q60932	VDAC1	32351	31427	8,55	7,50	1	19	42	Mouse
Vinculin	P18206	VINC a	123799	119845	5,5	6,16	1	16	26	Human homolog
Vinculin	P18206	VINC b	123799	119845	5,5	6,24	1	25	30	Human homolog

1. Peptide Mass Fingerprint; 2. Nt-microsequencing; 3. Western Blot.
